# Supplementary material for: 1,2-Diarylethanols—A New Class of Compounds That Are Toxic to E. coli K12, R2–R4 Strains
Source: Materials (Basel). 2021 Feb 22;14(4):1025. doi: 10.3390/ma14041025 (PMC7926326; doi:10.3390/ma14041025)
Supplement: Supplementary file 1 [file materials-14-01025-s001.pdf]

materials

MDPI

Supplementary Materials

1,2-Diarylethanols—A New Class of Compounds That Are Toxic to *E. coli* K12, R2-R4 Strains

Paweł Kowalczyk [1,*], Damian Trzepizur [2], Mateusz Szymczak [3], Grzegorz Skiba [1], Karol Kramkowski [4] and Ryszard Ostaszewski [2]

Citation: Kowalczyk, P.; Trzepizur, D.; Szymczak, M.; Skiba, G.; Kramkowski, K.; Ostaszewski, R. 1,2-Diarylethanols—A New Class of Compounds That are Toxic to E. coli K12, R2-R4 Strains. *Materials* **2021**, *14*, 1025. https://doi.org/10.3390/ma14041025

Academic Editor: Lifeng Yan

Received: 29 January 2021
Accepted: 18 February 2021
Published: 22 February 2021

Publisher's Note: MDPI stays neutral with regard to jurisdictional claims in published maps and institutional affiliations.

Copyright: © 2020 by the authors. Submitted for possible open access publication under the terms and conditions of the Creative Commons Attribution (CC BY) license (http://creativecommons.org/licenses/by/4.0/).

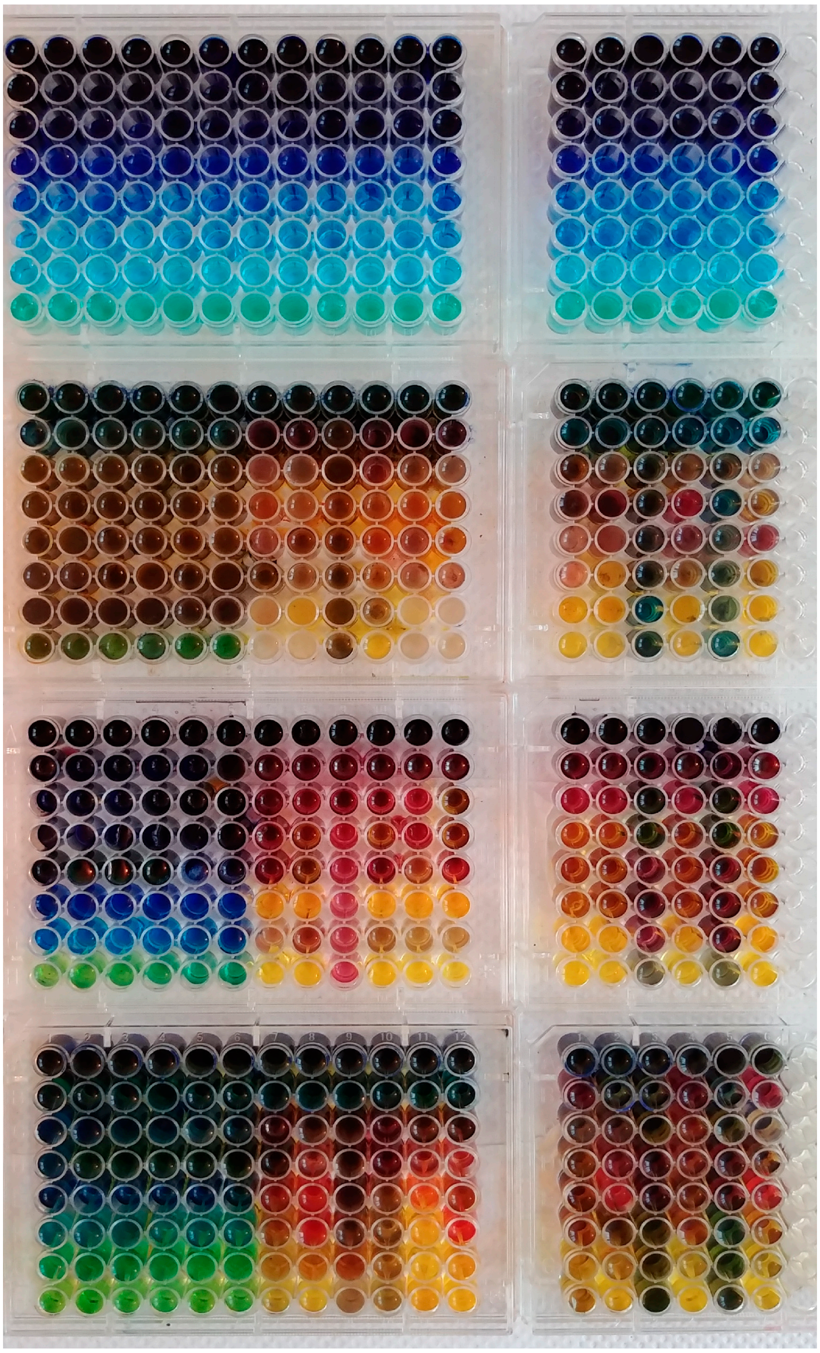

Figure S1. Examples of MIC on microplates with different concentration of studied compounds (mg L^{-1}). Resazurin was added as an indicator of microbial growth. Panel A–K12 strains, Panel B-

R2 strains, Panel C-R3 strains, Panel D- R4 strains with tested first 18 compounds. Wells 1 through 18 contain consecutive compounds as described in Table 1.

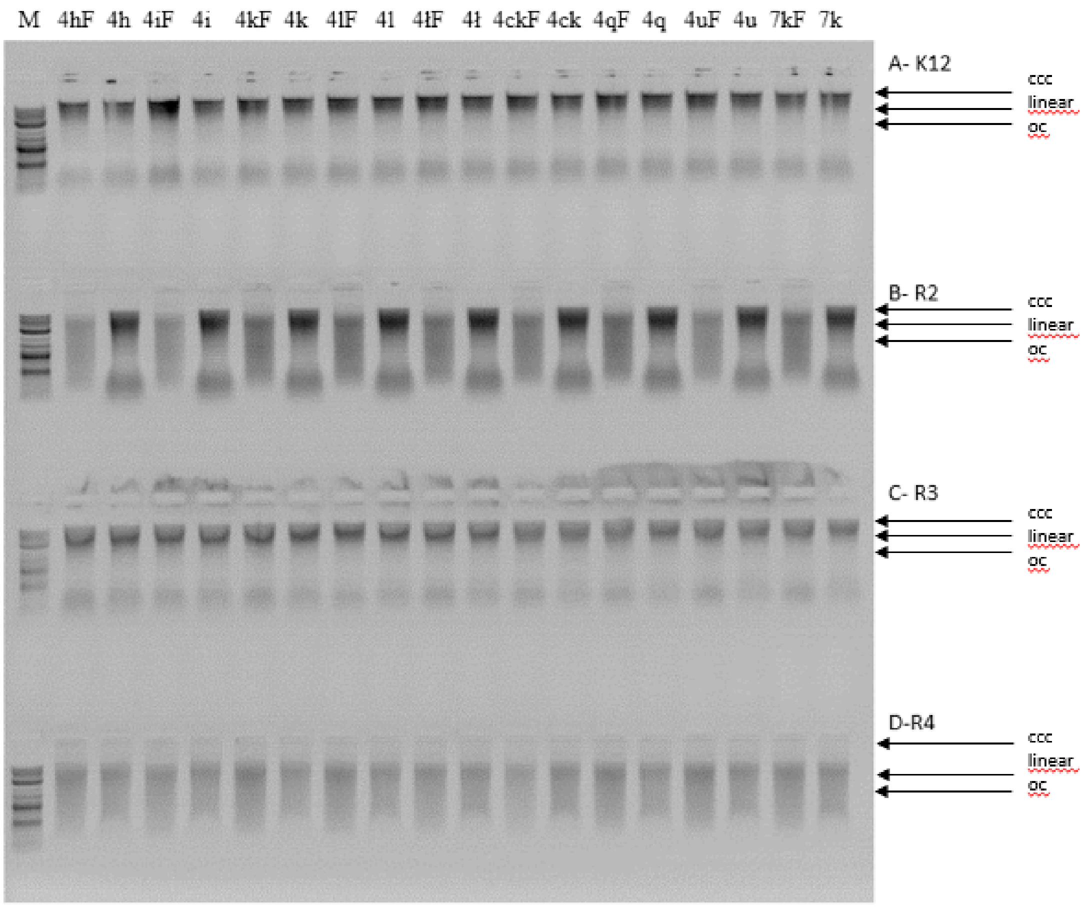

Figure S2. An example of an agarose gel electrophoresis separation of isolated plasmids DNA from K12 and R4 strains unmodified and modified with selected diaryloalcohols solution and digested (or not) with repair enzymes Fpg. Lanes: 1-18 (Panel A- control K12 strain), (panel B- R2 strain), (panel C- R3 strain), (panel D- R4 strain), F- plasmid digested with Fpg protein.

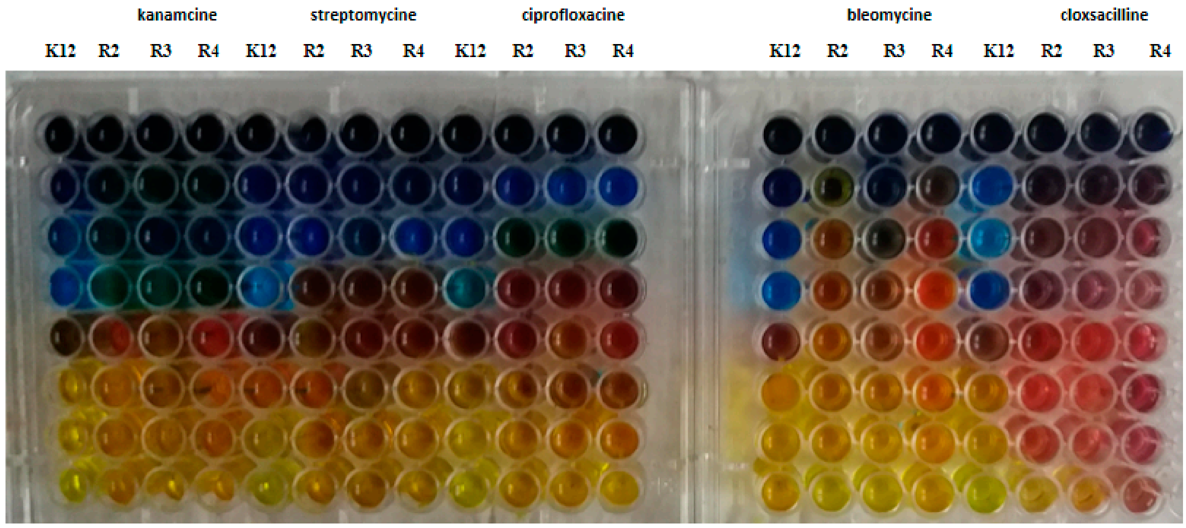

Figure S3. Examples of MIC with different strains K12, R2, R3, R4 of studied antibiotics with kanamycine, streptomycine, ciprofloxacine, bleomycine, cloxacilline.

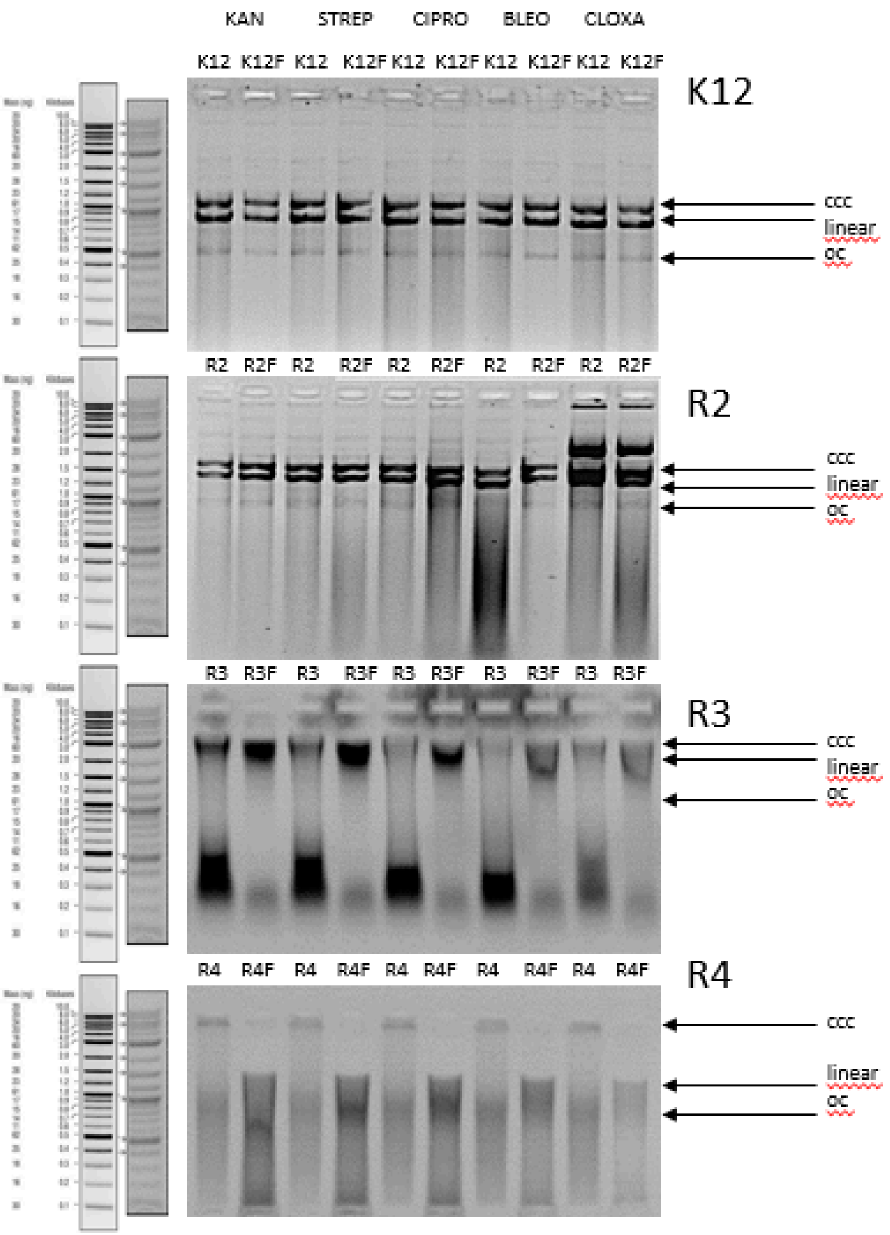

Figure S4. An example of an agarose gel electrophoresis separation of isolated plasmids DNA from K12, R2, R4 and R4 strains modified with antibiotics : kanamcine, streptomycine, ciprofloxacine, bleomycine and cloxacilline digested (or not) with repair enzymes Fpg.
